# *Hypericum perforatum* and Its Ingredients Hypericin and Pseudohypericin Demonstrate an Antiviral Activity against SARS-CoV-2

**DOI:** 10.3390/ph15050530

**Published:** 2022-04-25

**Authors:** Fakry F. Mohamed, Darisuren Anhlan, Michael Schöfbänker, André Schreiber, Nica Classen, Andreas Hensel, Georg Hempel, Wolfgang Scholz, Joachim Kühn, Eike R. Hrincius, Stephan Ludwig

**Affiliations:** 1 Institute of Virology Muenster, Center for Molecular Biology of Inflammation (ZMBE), University Hospital Muenster, 48149 Muenster, Germany; framadan@uni-muenster.de (F.F.M.); anhlan@uni-muenster.de (D.A.); m_scho79@uni-muenster.de (M.S.); andre.schreiber@uni-muenster.de (A.S.); hrincius@uni-muenster.de (E.R.H.); 2 Institute of Pharmaceutical Biology and Phytochemistry, University of Muenster, 48149 Muenster, Germany; n_clas01@uni-muenster.de (N.C.); ahensel@uni-muenster.de (A.H.); 3 Division of Clinical Pharmacy, Institute of Pharmaceutical and Medical Chemistry, University of Muenster, 48149 Muenster, Germany; georg.hempel@uni-muenster.de; 4 Hirsch Apotheke, 58507 Luedenscheid, Germany; wscholz@scholzdatabank.com; 5 Division of Clinical Virology, Institute of Virology, University Hospital Muenster, 48151 Muenster, Germany; kuehnj@uni-muenster.de; 6 Department of Virology, Faculty of Veterinary Medicine, Zagazig University, Zagazig 44511, Sharkia, Egypt

**Keywords:** COVID, SARS-CoV-2, coronavirus, plant extract, medicinal plants, antivirals, *Hypericum perforatum*, hypericin, pseudohypericin

## Abstract

For almost two years, the COVID-19 pandemic has constituted a major challenge to human health, particularly due to the lack of efficient antivirals to be used against the virus during routine treatment interventions. Multiple treatment options have been investigated for their potential inhibitory effect on SARS-CoV-2. Natural products, such as plant extracts, may be a promising option, as they have shown an antiviral activity against other viruses in the past. Here, a quantified extract of *Hypericum perforatum* was tested and found to possess a potent antiviral activity against SARS-CoV-2. The antiviral potency of the extract could be attributed to the naphtodianthrones hypericin and pseudohypericin, in contrast to other tested ingredients of the plant material, which did not show any antiviral activity. *Hypericum perforatum* and its main active ingredient hypericin were also effective against different SARS-CoV-2 variants (Alpha, Beta, Delta, and Omicron). Concerning its mechanism of action, evidence was obtained that *Hypericum perforatum* and hypericin may hold a direct virus-blocking effect against SARS-CoV-2 virus particles. Taken together, the presented data clearly emphasize the promising antiviral activity of *Hypericum perforatum* and its active ingredients against SARS-CoV-2 infections.

## 1. Introduction

Coronaviruses continuously circulate among humans, animals, and birds, with high zoonotic potential. At least three different coronaviruses had caused major public health threats in the last two decades, including the severe acute respiratory syndrome coronavirus-1 (SARS-CoV-1; discovered in 2002 in China) [1], the Middle East respiratory syndrome coronavirus (MERS-CoV; first identified in 2012 in Saudi Arabia) [2], and recently SARS-CoV-2 (emerged in late December 2019 in the city of Wuhan, China) [3]. The latter one caused a communicable disease called coronavirus disease-19 (COVID-19), which is a typical respiratory illness but in severe cases also causes pneumonia, cytokine dysregulation, multi-organ failure, and potential death [4]. Shortly after its emergence, the World Health Organization (WHO) announced the COVID-19 outbreak as a global pandemic. To date, COVID-19 cases have exceeded 503 million infections worldwide with more than 6.2 million deaths (updated in April 2022), illustrating the high morbidity and mortality rate and the rapid transmission of the virus. While effective vaccination strategies were developed in record time, the fight against the virus is hindered by the emergence of virus variants with higher transmission rates and immune-evasive properties.

SARS-CoV-2 is a single-stranded, positive-sense, enveloped RNA virus that belongs to the genus *Betacoronavirus* of the family *Coronaviridae* [5]. Members of this genus (SARS-CoV-1, SARS-CoV-2, MERS-CoV, and human coronaviruses NL63 and 229E, etc.) share a relatively similar genomic structure (about 30kb in length with high sequence homology), where the structural proteins, such as the spike (S), envelope (E), membrane (M), and nucleocapsid (N), are of major significance [6]. The E and M proteins are mainly involved in the virus assembly [7,8]. The viral RNA is encapsidated by the N protein, forming the viral ribonucleoprotein [9]. SARS-CoV-2 relies on its S protein for the attachment and entry of the virus into the host cell. The receptor-binding domain (RBD) in the S1 subunit of S protein recognizes and binds to the human angiotensin-converting enzyme 2 (hACE2) receptors on the surface of airway epithelial cells. To further fuse with cellular membranes, the SARS-CoV-2 S protein must be proteolytically cleaved, which could be achieved by cellular proteases, such as the cell-surface transmembrane serine protease 2 (TMPRSS2) or the endosomal/ lysosomal cathepsins or furin-like enzymes. Once uncoating is achieved, the replication of the viral +ssRNA is directly initiated inside the infected cells. The virus entry features of SARS-CoV-2, particularly at the S protein/ACE2 interface, contribute mainly to the rapid transmission and severity of the disease, as they directly affect infectivity, host-adaptation processes, and immune evasion [10,11,12].

Shortly after the beginning of the pandemic, distinct genetic lineages (variants) of the virus emerged and continued circulating across the world [13,14]. The European Centre for Disease Prevention and Control (ECDC) announced four categories for the emerging variants as of October 2021, which include (i) variants of concern (VOC), (ii) variants of interest (VOI), (iii) variants under monitoring/investigation, and (iv) de-escalated variants. The members of the VOC group showed a significant impact on transmissibility, severity, or immunity and involve the variants Beta (B.1.351), Gamma (P.1), Delta (B.1.617.2), and most recently the Omicron variant (B.1.1.529). On the other hand, de-escalated variants (e.g., the Alpha “B.1.1.7” variant) are those that are no longer circulating or had minimum and/or no epidemiological impact.

Several approaches were introduced to reveal potential antivirals against SARS-CoV-2. Among them, the first installed one was the usage of existing clinically approved drugs (i.e., re-purposing of drug usage) [15]. Nevertheless, undesired side effects may occur in terms of different doses, toxic effects, and pharmacological characteristics. The second approach is computational drug screening [16], which unfortunately needs extensive subsequent experimental validation. The third approach is the development of new drugs that could inhibit SARS-CoV-2, which, of course, is time-consuming and expensive. Along that line, another approach, that was introduced with many human and/or animal viruses and recently with SARS-CoV-2 as well, is to test the ability of natural plant-derived (herbal or medicinal) products to inhibit the virus at certain points of its entry and/or early replication cycle [17]. Multiple plant-originated compounds were tested for their antiviral potential as well as treatment of some medical disorders [18]. It is worth mentioning that targeting virus entry or virus stability/survival in the environment will result in the prevention of infection and transmission among individuals. Accordingly, such antivirals could be used as prophylactic and/or therapeutic agents. It was proposed that direct physical interaction between the selected plant extract and the target virus can cause (i) virion destruction, (ii) disruption of surface proteins of the virus, (iii) interference with the virus adsorption/attachment, (iv) blockage of virus penetration/internalization into susceptible host cells, or (v) cessation of early virus-replication events. Such early actions protect the cells from virus invasion and eventually can result in abortion of viral infection [19,20].

Here, a quantified plant extract of *Hypericum perforatum* was investigated for its antiviral activity against SARS-CoV-2. *Hypericum perforatum*, also known as St. John’s wort, is a widespread herbal plant, which is involved in many therapeutic applications, as it possesses (i) anti-depressant, (ii) anti-cancer, (iii) anti-oxidative and neuro-protective, (iv) wound-healing, (v) anti-inflammatory, and (iv) antimicrobial properties [21]. *Hypericum perforatum*, monographed for quality reasons also in the European Pharmacopoeia for medicinal use, contains high amounts of condensed tannins from the procyanidin group in addition to flavonoids, phloroglucinols, and naphtodianthrones. Several reports on potential antiviral activities of the extract or its active ingredients, such as the naphtodianthrone hypericin, were published. In these studies, these antiviral activities were shown against herpes simplex virus type 1 [22], human cytomegalovirus [23], hepatitis B virus [24], influenza A virus [25,26], human immunodeficiency virus type 1 [27], and animal viruses [28,29], including the infectious bronchitis virus and the porcine epidemic diarrhea virus, as gamma- and alpha- coronaviruses, respectively [30,31].

Here, by using a pseudo-typed vesicular stomatitis virus (VSV) that harbors the SARS-CoV-2 S protein as its surface protein, the capability of a quantified *Hypericum perforatum*, as well as its naphtodianthrone hypericin and pseudohypericin, to block the SARS-CoV-2 S-mediated entry was demonstrated, indicating an interference of the compounds at the early attachment or entry phase of the virus. The antiviral activity of these compounds was further tested and fully confirmed against the genuine SARS-CoV-2 and emerging virus variants.

## 2. Results

### 2.1. The Hypericum perforatum Extract (HP1) Inhibits Infection of Cells by the Pseudo-Typed VSV SARS-CoV-2 S Protein-d21-Carrying Virus

To test its potential antiviral activity against SARS-CoV-2, a quantified dry extract (HP1) from the flowering parts of *Hypericum perforatum* L. was prepared by methanol-water extraction and subsequent drying. The extract complied to the specification of the European Pharmacopoeia (Ed. 10). Detailed quantitative data of HP1 are displayed in the Materials and Methods section. Additionally, LC-DAD-ESI-qTOF-MS in positive polarity was used for detailed extract characterization and fingerprinting. Peaks to identify were selected from UV chromatograms at λ = 360 and 275 nm, according to the assay on hyperforin and flavonoids, as found in Ph. Eur. 10.0, monograph “St. John’s wort dry extract, quantified” (Figure S1). All the expected compounds resembled the pattern of the exemplified chromatograms shown in the EDQM knowledge database [32]. Other peaks of comparable intensity were also identified as known compounds of *Hypericum perforatum* by their accurate masses [33]. All identified compounds from detailed peak dereplication data are displayed in the Table S1.

To evaluate suitable non-toxic doses for antiviral testing, potential cell toxicities of the HP1 extract were first tested on Vero cells by using the MTT-based cytotoxicity assay. Initially, it was revealed that concentrations up to 50 µg/mL of HP1 did not show any cell toxicity (Figure S2A). After revealing tolerance of Vero cells to such concentrations, the anti-SARS-CoV-2 activity of HP1 (50 µg/mL) was tested by using a VSV pseudo-typed virus system carrying the SARS-CoV-2 S protein as its surface protein. This system allows the analysis of SARS-CoV-2 S protein-mediated attachment and entry. Accordingly, testing selected compounds could reveal a potential interference with this process. Analyzing the inhibitory potential of HP1 against SARS-CoV-2 S protein-mediated virus infection revealed that 50 µg/mL of HP1 reduced the number of infected cells by nearly 57% in comparison to the control (Figure S2B). Based on this promising antiviral activity of the HP1 extract against the VSV pseudo-typed SARS-CoV-2 S protein-carrying virus, the investigations were further expanded by deeply analyzing the inhibitory concentration 50 (IC_50_) and cytotoxic concentration 50 (CC_50_).

**Figure 1:**
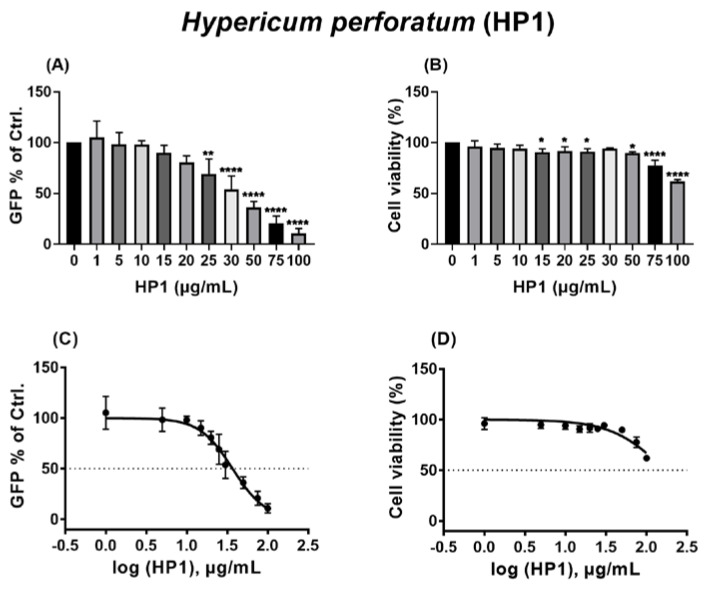
The antiviral activity of *Hypericum perforatum* (HP1) against the pseudo-typed VSV virus carrying the SARS-CoV-2 S protein. (**A**,**C**) Vero cells were seeded overnight, and on the next day, cells and the VSV-pseudo-typed virus were incubated with *Hypericum perforatum* (HP1) or solvent control (DMSO) for 1 h prior to infection, at 37 °C or room temperature, respectively. After pre-incubation, infection was performed with a MOI of 0.01 for 1 h, and cells were finally washed and incubated without further treatments. After 16–18 h, GFP signal was visualized under fluorescent microscope. (**A**) GFP-positive cells as % of control are shown (mean and s.d.), and one-way ANOVA with Dunnett’s multiple comparisons was done by comparing each value with the control. (**C**) Dose–response curve of the normalized GFP-positive cell values as % of control is depicted (mean and s.d.). (**B**,**D**) Vero cells were seeded overnight, and on the next day, incubation with HP1 or solvent control was initiated. 24 h after the start of incubation, the MTT assay-based cytotoxicity was measured. (**B**) Cell viability as % of control is shown (mean and s.d.), and one-way ANOVA with Dunnett’s multiple comparisons was done by comparing each value with the control. (**D**) Dose–response curve of the normalized cytotoxicity values as % of control is depicted (mean and s.d.). * for *p* ≤ 0.05, ** for *p* ≤ 0.01, and **** for *p* ≤ 0.0001.

A wide range of HP1 concentrations (from 1 to 100 µg/mL) was selected for testing. As displayed in Figure 1, it was shown that HP1 started to inhibit the pseudo-typed virus at concentrations of as low as 15 µg/mL, and the inhibitory effect increased gradually with increasing amounts of the extract, as 50 µg/mL reduced the number of infected cells by more than 50% compared to the control, as evidenced by expression of the marker protein GFP (Figure 1A). The same selected concentrations were submitted to MTT assay-based cell toxicity analyses, which showed increased cell toxicities from 75 µg/mL (Figure 1B). When the obtained data were used for dose–response curves and IC_50_ and CC_50_ analyses (Figure 1C,D), the IC_50_ of HP1 was 36.88 µg/mL (Figure 1C and Table S2). The highest tested concentration of HP1 did not result in 50 % cytotoxicity (Figure 1D, Table S2). Therefore, the CC_50_ of the extract is at least higher than 100 µg/mL (Table S2).

In summary, the HP1 extract acts as a strong antiviral agent against the VSV pseudo-typed SARS-CoV-2 S protein-carrying virus.

### 2.2. The Naphtodianthrones Hypericin and Pseudohypericin from HP1 Are Active against the Pseudo-Typed VSV SARS-CoV-2 S Protein-d21-Carrying Virus

**Figure 2:**
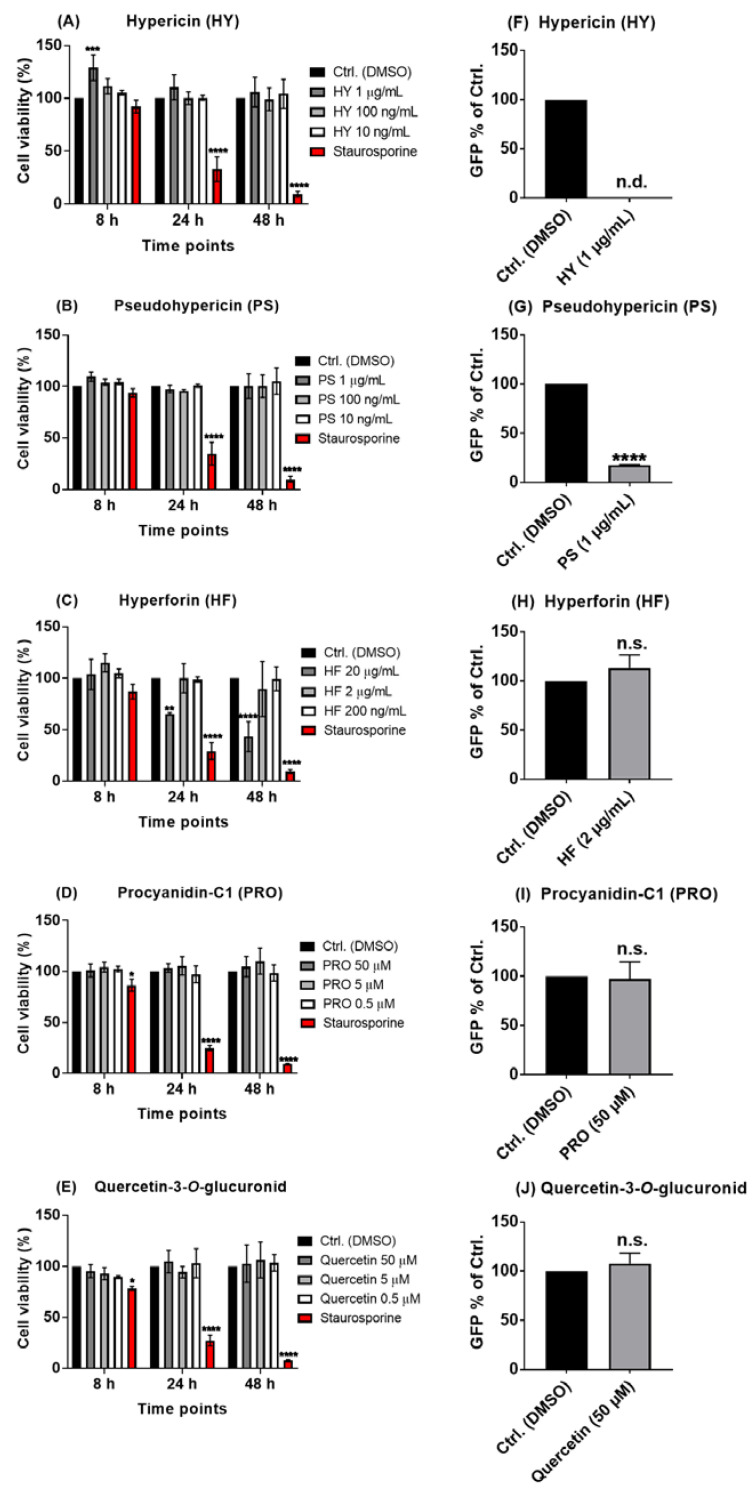
Hypericin and pseudohypericin are key components in the *Hypericum perforatum* (HP1) extract that are antivirally effective against the pseudo-typed VSV virus. (**A**–**E**) Vero cells were seeded overnight, and the next day, different concentrations of hypericin (HY), pseudohypericin (PS), hyperforin (HF), procyanidin-C1 (PRO), and quercetin-3-*O*-glucuronid (ingredients of HP1 extract) were applied onto the cells for indicated time points, as solvent-treated cells (DMSO) served as control. In addition, Staurosporine-treated cells served as positive control. After the incubations, the MTT assay-based cytotoxicity was measured, cell viability as % of solvent control is shown (mean and s.d), and two-way ANOVA with Dunnett’s Multiple comparisons was done by comparing each value with the solvent control at each time point. (**F**–**J**) Vero cells were seeded overnight, and on the next day, cells and the VSV-pseudo-typed virus were incubated with the indicated substances or solvent control (DMSO) for 1 h prior to infection, at 37 °C or room temperature, respectively. After the pre-incubation, infection was performed with a MOI of 0.01 for 1 h, and cells were finally washed and incubated without further treatments. GFP-positive cells as % of solvent control are shown (mean and s.d.), and Student’s *t*-test with Welch’s correction was applied (n.d. means non-detected, while n.s. means non-significant statistical difference). * for *p* ≤ 0.05, ** for *p* ≤ 0.01, *** for *p* ≤ 0.001, and **** for *p* ≤ 0.0001.

Since plant extracts, such as *Hypericum perforatum*, are a complex mixture of multiple secondary products, a systematic investigation of isolated chemically defined ingredients of HP1 was performed in order to pinpoint the potential antiviral compounds. Based on the known composition of *Hypericum perforatum*, five major components of the extract, namely the two naphtodianthrones hypericin and pseudohypericin, the phloroglucinol derivative hyperforin, the proanthocyanidin/condensed tannins procyanidin C1, and the flavonol glycoside (quercetin-3-*O*-glucuronid) (Figure S3), were investigated for their potential antiviral capacity in the VSV pseudo-typed virus system. Again, for each compound, MTT assay-based cytotoxicity measurements were performed on Vero cells. Hypericin and pseudohypericin did not show any influence on cell viability up to 1 µg/mL (Figure 2A,B), and hyperforin showed no toxicity until 2 µg/mL but was toxic at a concentration of 20 µg/mL (Figure 2C). Procyanidin-C1 and quercetin-3-*O*-glucuronid were not toxic up to 50 µM (Figure 2D,E). 

When testing these five ingredients against the pseudo-typed VSV SARS-CoV-2 S protein-carrying virus, hypericin completely blocked the infection by the pseudo-typed virus (Figure 2F), while pseudohypericin reduced it by nearly 82% compared to the solvent control (Figure 2G). In contrast, hyperforin, procyanidin-C1, and quercetin-3-*O*-glucuronid did not have any noticeable impact on the ability of the pseudo-typed virus particles to infect cells (Figure 2H–J).

These findings illustrate that hypericin and pseudohypericin may be the main compounds of the *Hypericum perforatum* extract that are responsible for its antiviral activities against SARS-CoV-2.

### 2.3. Hypericin and Pseudohypericin Exhibit a Strong Antiviral Activity against the Pseudo-Typed VSV SARS-CoV-2 S Protein-d21-Carrying Virus

**Figure 3:**
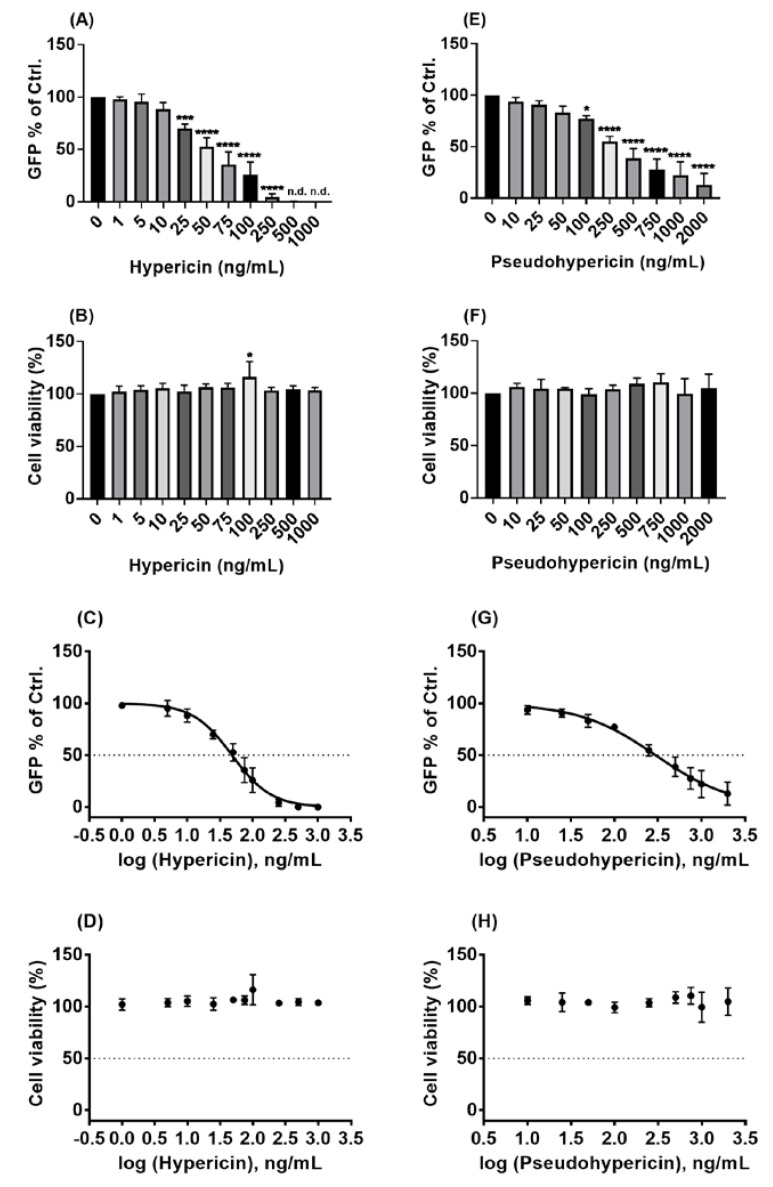
Hypericin and pseudohypericin showed a strong antiviral activity against the pseudo-typed VSV virus. (**A**,**C**,**E**,**G**) Vero cells were seeded overnight, and the next day, prior to infection (MOI = 0.01), both cells and the VSV pseudo-typed virus were incubated with the indicated substances or solvent control (DMSO) for 1 h, at 37 °C and room temperature, respectively. After the pre-incubation, cells were infected for 1h and finally washed and incubated without further treatments. (**A**,**E**) GFP-positive cells as % of solvent control are shown (mean and s.d.), and one-way ANOVA with Dunnett’s multiple comparisons was done by comparing each value with the control. (**B**,**D**,**F**,**H**) Vero cells were seeded overnight, and the next day, incubation with the indicated substances or solvent control (DMSO) was initiated. After the 24 h incubation, MTT assay-based cytotoxicity was measured. (**B**,**F**) Cell viability as % of solvent control is shown (mean and s.d.), and one-way ANOVA with Dunnett’s multiple comparisons was done by comparing each value with the solvent control. (**C**,**G**) Dose–response curve of the normalized GFP-positive cell values as % of solvent control is depicted (mean and s.d.). (**D**,**H**) Dose–response curve of the normalized cytotoxicity values as % of solvent control is depicted (mean and s.d.). n.d means non-detected, * for *p* ≤ 0.05, *** for *p* ≤ 0.001, and **** for *p* ≤ 0.0001.

As hypericin and pseudohypericin were found to have the strongest antiviral activity against the pseudo-typed virus in the first pilot assays, detailed IC_50_ and CC_50_ analyses for both compounds were conducted by examining a broad range of different concentrations. As shown in Figure 3A, the inhibiting effects of hypericin on the pseudo-typed virus were clearly observed, where 50 ng/mL resulted in about 50% reduction, and the concentration of 500 ng/mL or higher did completely block virus infection. Testing for cytotoxicity revealed that the concentrations of hypericin (up to 1000 ng/mL) are not toxic for Vero cells (Figure 3B). Using the obtained data for dose–response analyses resulted in an IC_50_ value of 48.5 ng/mL (96 pmol/mL) for hypericin against the pseudo-typed virus (Figure 3C, Table S2). This very low IC_50_ stands against a high CC_50_ of > 1000 ng/mL, indicating a broad therapeutic window of the compound with a selectivity index (SI) of > 20 (Figure 3D, Table S2). Analyzing IC_50_ and CC_50_ for pseudohypericin, a concentration of 250 ng/mL (480 pmol/mL) was required to result in a roughly 50% reduction of infected cells (Figure 3E). Pseudohypericin was also not toxic to Vero cells in the tested concentrations (up to 2000 ng/mL), as the viability of Vero cells was completely unchanged (Figure 3F). For pseudohypericin, calculations revealed an IC_50_ of 298.4 ng/mL (573 pmol/mL) (Figure 3G, Table S2) and a CC_50_ of at least 2000 ng/mL (Figure 3H, Table S2).

Taken together, the two naphtodianthrones, namely hypericin and pseudohypericin, of HP1 are strongly active against the used VSV pseudo-typed virus in non-toxic concentrations.

### 2.4. Hypericum perforatum (HP1) and Its Ingredients, Hypericin and Pseudohypericin, Are Antivirally Active against SARS-CoV-2

The antiviral activity of HP1 and its ingredients, hypericin and pseudohypericin, that has been shown so far against the VSV pseudo-typed virus was further confirmed against genuine infectious SARS-CoV-2 virus. For these investigations, an experimental treatment protocol, including pre-treatment of both cells and virus prior to infection and further post-treatment of cells after SARS-CoV-2 infection, was applied.

**Figure 4:**
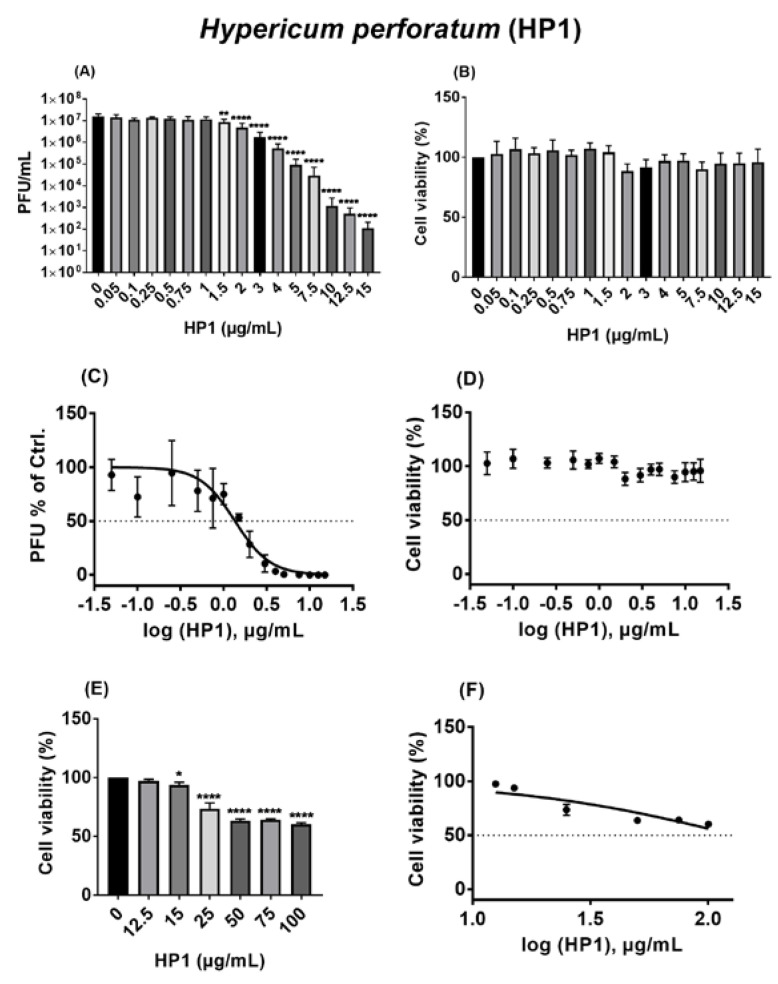
*Hypericum perforatum* (HP1) acts as an antiviral against ancestral SARS-CoV-2. (**A**,**C**) Vero cells were seeded overnight, and on the next day, prior to infection (MOI = 0.05), cells were incubated at 37 °C for 1h with infection-DMEM containing either solvent control (DMSO) or HP1. Concurrently, SARS-CoV-2 was incubated for 1 h at room temperature in infection-PBS that contained either DMSO or HP1. After infection (37 °C/1 h), cells were further incubated in infection-DMEM including either DMSO or HP1. After 24 h, virus supernatants were collected and subjected to plaque assay. (**A**) Results are expressed as PFU/mL (mean and s.d.), and one-way ANOVA with Dunnett’s multiple comparisons was done by comparing each value with the control. (**C**) Dose–response curve of the normalized virus titer values as % of solvent control is depicted (mean and s.d.). (**B**,**D**–**F**) Vero cells were seeded overnight, and on the next day, cells were incubated for 24 h with infection-DMEM that contained either solvent control (DMSO) or HP1. After incubation, the MTT assay-based cytotoxicity was measured. (**B**,**E**) Cell viability as % of solvent control is shown (mean and s.d.), and one-way ANOVA with Dunnett’s multiple comparisons was done by comparing each value with the control. (**D**,**F**) Dose–response curve of the normalized cytotoxicity values as % of solvent control is depicted (mean and s.d.). * for *p* ≤ 0.05, ** for *p* ≤ 0.01, and **** for *p* ≤ 0.0001.

For HP1, concentrations from 0.05 to 15 µg/mL were tested against SARS-CoV-2, where those from 0.05 to 1 µg/mL had no considerable effect on virus replication. Progeny virus amounts started to drop significantly from 1.5 µg/mL, and the highest tested concentration of HP1 (15 µg/mL) caused a decline of multiple log steps in virus titers (Figure 4A). Evaluating the potential toxicity of the used concentrations showed that they were non-toxic to Vero cells, based on the obtained MTT results (Figure 4B). Using the obtained data sets for dose–response analyses, the IC_50_ of HP1 was 1.35 µg/mL (2.68 nmol/mL) (Figure 4C, Table S2). Due to the non-toxic nature of concentrations up to 15 µg/mL, that have been tested so far, the CC_50_ value could not be determined (Figure 4D, Table S2). To further determine the maximum tolerated concentrations, a dose-escalation toxicity assay of HP1 with concentrations up to 100 µg/mL was conducted, which clarified that cell toxicities can be clearly seen starting from 25 µg/mL, with close to 50% cell toxicity by using 100 µg/mL of HP1 (Figure 4E). Using this data set for dose–response analyses for CC_50_ values showed that the CC_50_ is at least higher than 100 µg/mL (Figure 4F, Table S2).

Experimental analysis of the antiviral activity of the HP1 ingredient hypericin against the SARS-CoV-2 showed virus-blocking activities starting from a concentration of 500 pg/mL (Figure S4A). Increasing hypericin concentrations resulted in a stronger reduction of virus titer, where hypericin entirely blocked SARS-CoV-2 virus propagation at concentrations of 25,000 and 50,000 pg/mL (Figure S4A). Within this range of tested concentrations, no obvious cytotoxic effects were observed (Figure S4B). Using the obtained data sets for IC_50_ calculations, the IC_50_ of hypericin against SARS-CoV-2 was 559 pg/mL (1.11 pmol/mL) (Figure S4C, Table S2), and the CC_50_ was higher than the maximal tested concentration of 50,000 pg/mL (Figure S4D, Table S2). To reveal the CC_50_ value for hypericin, dose escalations were performed by determining the associated cell toxicity with concentrations between 0.025 and 100 µg/mL, where none of them showed substantial toxic effects on Vero cells (Figure S4E), confirming that the CC_50_ of hypericin is higher than 100 µg/mL (Figure S4F, Table S2).

Similar to the antiviral activities of hypericin against SARS-CoV-2, pseudohypericin (differing only in one hydroxylation moiety compared to hypericin) was also active in inhibiting SARS-CoV-2 infection. As a wide concentrations range (1–1,000,000 pg/mL) was tested, the antiviral effect was seen starting from 25,000 pg/mL, and 1,000,000 pg/mL totally blocked virus infection (Figure S5A). The MTT assay-based analysis showed no cell toxicity to Vero cells (Figure S5B). Based on that, pseudohypericin had an IC_50_ of 20,036 pg/mL (38.5 pmol/mL) (Figure S5C, Table S2). However, as the CC50 was higher than 1,000,000 pg/mL (Figure S5D), a dose-escalation trial was conducted to investigate the CC_50_ value of pseudohypericin. By applying concentrations between 0.5 and 10 µg/mL on cells, no toxicity was observed (Figure S5E). Therefore, the CC_50_ of pseudohypericin was confirmed to be higher than 10 µg/mL (Figure S5F, Table S2).

Taken together, *Hypericum perforatum* and its ingredients, namely hypericin and pseudohypericin, are antivirally active against SARS-CoV-2. For all three substances, multiple log-step reductions in virus titer could be observed with concentrations proven to be non-toxic for the tested host cell.

### 2.5. Hypericum perforatum (HP1) and Hypericin Displayed an Antiviral Activity against SARS-CoV-2 Variants

**Figure 5:**
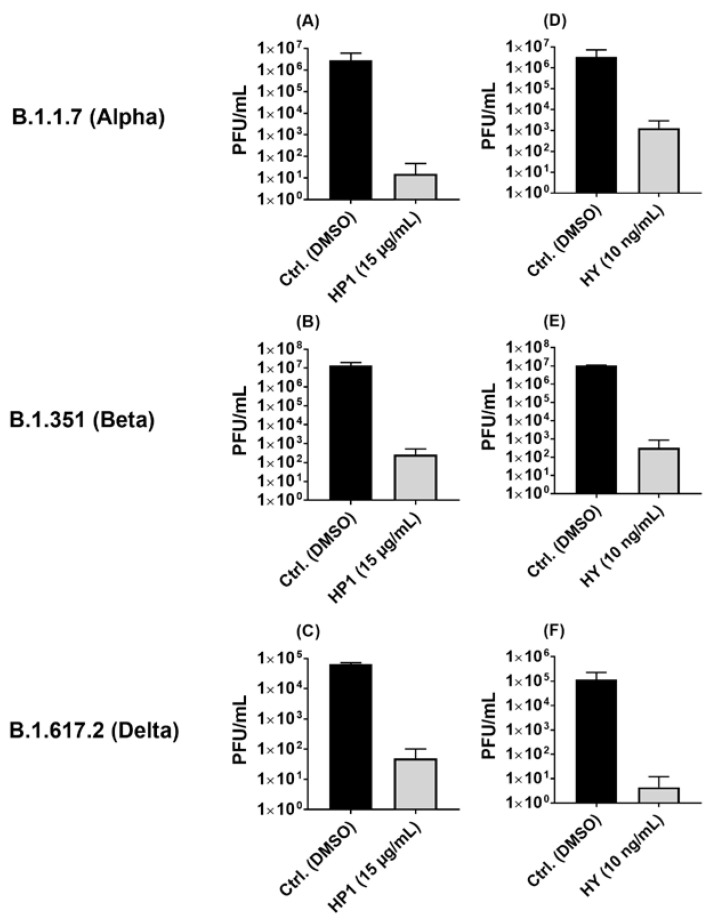
*Hypericum perforatum* (HP1) and hypericin (HY) inhibit the growth of different SARS-CoV-2 variants. (**A**–**F**) Vero cells were seeded overnight, and the next day, before being infected, cells were incubated in infection-DMEM containing solvent control (DMSO) or either (**A**–**C**) HP1 or (**D**–**F**) hypericin for 1h, at 37 °C. Meanwhile, SARS-CoV-2 variants were also preincubated (1 h at room temperature) before infection in infection-PBS with either solvent control or (**A**–**C**) HP1 or (**D**–**F**) hypericin. After pre-incubation, virus infection was performed at a MOI of 0.05 for 1 h. After infection, cells were either incubated with solvent control or (**A**–**C**) HP1 or (**D**–**F**) hypericin. After 24 h infection, virus supernatants were collected, and virus titration was done by plaque assays. (**A**–**F**) Obtained data are shown as PFU/mL (mean and s.d.).

As HP1 and its most active ingredient, hypericin, demonstrated a potent antiviral activity against ancestral SARS-CoV-2, emerged SARS-CoV-2 variants were included for further investigation. Accordingly, the antiviral activity of the HP1 extract and hypericin was investigated against three well-known SARS-CoV-2 variants (Alpha “B.1.1.7”, Beta “B.1.351”, and Delta “B.1.617.2”). In correlation to the solvent control, HP1 treatment was shown to reduce the virus titers of the Alpha, Beta, and Delta variants by multiple log steps (Figure 5A–C). Accordingly, hypericin treatment also caused a drop in the SARS-CoV-2 titers of all three variants by multiple log steps (Figure 5D–F).

In summary, *Hypericum perforatum* and hypericin are also active against key emerged SARS-CoV-2 variants, pointing out that their robust mode of action is not affected by SARS-CoV-2 variants mutations.

### 2.6. Pre-Treatment of SARS-CoV-2 Virus Particles Prior to Infection with Hypericum perforatum (HP1) or Hypericin Is Mostly Effective in Blocking Virus Infection

**Figure 6:**
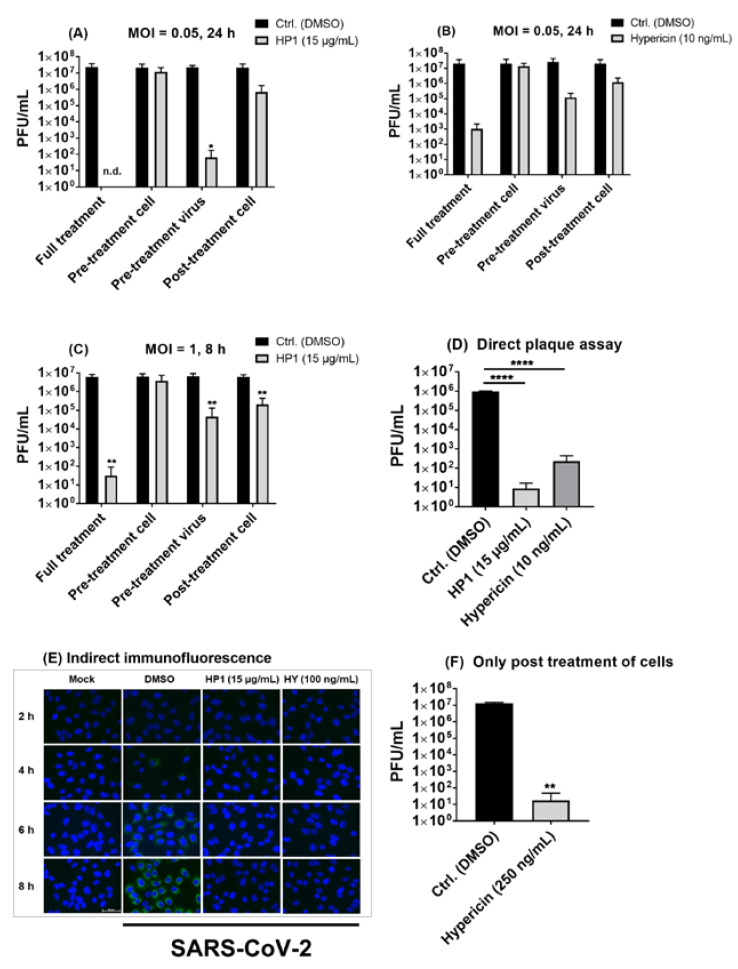
*Hypericum perforatum* (HP1) and hypericin (HY) carry direct SARS-CoV-2 virus-blocking activities. (**A**–**C**) Vero cells were seeded overnight. The next day, different treatment protocols with HP1 or hypericin were applied. The treatment conditions included (i) only pre-treatment of cells (for 1 h at 37 °C) in infection-DMEM containing solvent control (DMSO) or HP1 or hypericin, (ii) only pre-treatment of SARS-CoV-2 (for 1 h at room temperature) in infection-PBS containing solvent control or HP1 or hypericin, or (iii) only post-treatment of cells after infection in infection-DMEM (at 37 °C) containing solvent control or HP1 or hypericin. As control, the combined treatment protocol of pre-treatment of cells and SARS-CoV-2 and post-treatment of cells was included as well (Full treatment). The SARS-CoV-2 infection was conducted at MOI of 0.05 or 1, as the total length of the infection experiment was (**A**,**B**) 24 h or (**C**) 8 h, respectively. (**A**–**C**) After the depicted length of experiments, virus supernatants were harvested, virus titration was done with plaque assay, results are shown as PFU/mL (means and s.d.), and two-way ANOVA with Sidak’s multiple comparisons was done by comparing each value to its respective solvent control. (**D**) SARS-CoV-2 was incubated for 1 h at room temperature with solvent control (DMSO) or HP1 or hypericin in an infection-PBS mix and directly submitted to plaque assay. Obtained data are expressed as PFU/mL (mean and s.d.), and one-way ANOVA with Dunnett’s multiple comparisons was done by comparing each value to the solvent control. (**E**) After Vero cells were seeded on cover slips overnight, cells were infected with 1 h pre-treated (either with DMSO or 15 µg/mL HP1 or 100 ng/mL hypericin (HY)) SARS-CoV-2 virus. Mock-infected cells served as control. Then, 2, 4, 6, and 8 h after infection, cold methanol (–20 °c) was used for cell fixation, and indirect immunofluorescence staining of the SARS-CoV-2 nucleoprotein (green) and nuclei (blue) was conducted. Exposure times for each channel where fixed on the 8 h infected and DMSO-treated samples (scale bar represents 50µM). (**F**) The day after seeding, Vero cells were infected with SARS-CoV-2 (MOI = 0.05) for 1 h. After infection, cells were either incubated with solvent control or hypericin (250 ng/mL) in infection-DMEM. Supernatants were collected 24 h after infection and submitted to plaque assay. Obtained data are shown as PFU/mL, and Student’s *t*-test with Welch’s corrections was done. n.d means non-detected. * for *p* ≤ 0.05, ** for *p* ≤ 0.01, and **** for *p* ≤ 0.0001.

After clarifying the broad activity against SARS-CoV-2 and emerging variants, we aimed to gain insights into the putative mode of action of HP1 and hypericin against SARS-CoV-2. To shed some light on their respective mode of action, the full treatment condition (pre-treatment of cells and virus plus post-treatment of cells), which had been used so far for the testing of indicated substances against SARS-CoV-2, was divided into single treatments, such as only pre-treatment of cells, only pre-treatment of the virus, and only post-treatment of cells. For the multicycle (24 h)-infection time point, it was revealed that pre-treatment of SARS-CoV-2 virus (prior to infection of cells) with HP1 or hypericin resulted in a robust reduction in virus titers (Figure 6A,B). Furthermore, only post-treatment of cells with the compounds after infection resulted in an antiviral activity but to a lesser extent than in the virus pre-treatment scenario (Figure 6A,B). In contrast, pre-treatment-only of cells with HP1 or hypericin did not affect infection and propagation of SARS-CoV-2, compared to control conditions (Figure 6A,B). Of note, similar results were observed in an 8 h single-cycle infection protocol, where the strongest antiviral activity was again observed in pre-incubated virus samples, while no effect of pre-treatment of cells was observed (Figure 6C).

To further confirm the virus-infectivity blocking or even virucidal activity of HP1 and hypericin, SARS-CoV-2 virus was incubated with either one of the tested compounds as in the virus pre-treatment-only protocol. Afterwards, samples were directly diluted and submitted to plaque assay, where a multiple log-step decline in infectious virus titers in HP1- or hypericin-treated samples was observed (Figure 6D), which further indicates that both substances have a direct virus-blocking effect against SARS-CoV-2. Tracing SARS-CoV-2 protein expression over the course of virus replication by using immunofluorescence-staining techniques, pre-treatment-only of SARS-CoV-2 with HP1 (15 µg/mL) or hypericin (100 ng/mL) prior to infection resulted in a strong block of the expression of the SARS-CoV-2 nucleocapsid protein (N). At none of the analyzed time points, N expression could be detected after virus pre-incubation with HP1 or hypericin (Figure 6E). In contrast, solvent-treated control samples showed increasing N expression as early as 4 h and most prominently at 6–8 h post infection (Figure 6E), which further confirms the direct action of both compounds on the SARS-CoV-2 virus particle infectivity.

Due to the obtained putative mode of action of HP1 and its ingredient hypericin, as it was primarily affecting the infectivity of the virus, it was questioned if we could achieve substantial multiple log steps spanning virus-blocking activity with a post-infection-only treatment protocol as well. Therefore, a selected non-toxic higher concentration of hypericin (250 ng/mL) was used for post-treatment of cells 1 h after SARS-CoV-2 infection, which resulted in up to a 6-log-step reduction in virus titer compared to solvent control conditions (Figure 6F). This clarifies that hypericin is an effective and strong antiviral acting component against SARS-CoV-2, even if treatment is initiated after virus infection.

Taken together, we could conclude that *Hypericum perforatum* (HP1) and its ingredients hypericin possess a strong antiviral activity against SARS-CoV-2 and recently emerged virus variants by blocking very early steps of virus infection. In addition, start of treatment after SARS-CoV-2 infection did also clearly block virus growth, which does not exclude the possibility that these compounds may have an additional secondary intracellular activity.

### 2.7. The Antiviral Effect of Hypericum perforatum (HP1) and Hypericin Is Not Mediated by Blocking Specific SARS-CoV-2 S Protein Functions

For a deeper mechanistic understanding of the HP1 extract-mediated blockage of SARS-CoV-2 propagation, a virus-free hACE2-RBD sVNT assay was conducted to reveal if HP1 or its ingredient hypericin can block the binding of SARS-CoV-2 S protein RBD to ACE2 receptors. The obtained results indicate that neither HP1 nor hypericin inhibits the binding of the S protein RBD fragment to hACE2 (Figure S6A). In addition, a virus-free cell–cell fusion assay revealed that HP1 and hypericin did not show any pronounced effects on S protein-mediated cell fusion activity (Figure S6B).

Taken together, both outcomes of the cell fusion assay and the hACE2-RBD sVNT assay indicate that the antiviral effects of HP1 and hypericin likely do not depend on any interference with the SARS-CoV-2 spike protein that would result in loss of S protein functions needed for virus infection.

### 2.8. The Antiviral Activity of Hypericum perforatum (HP1) and Hypericin against the VSV Pseudo-Typed Virus Carrying the Omicron S Protein

**Figure 7:**
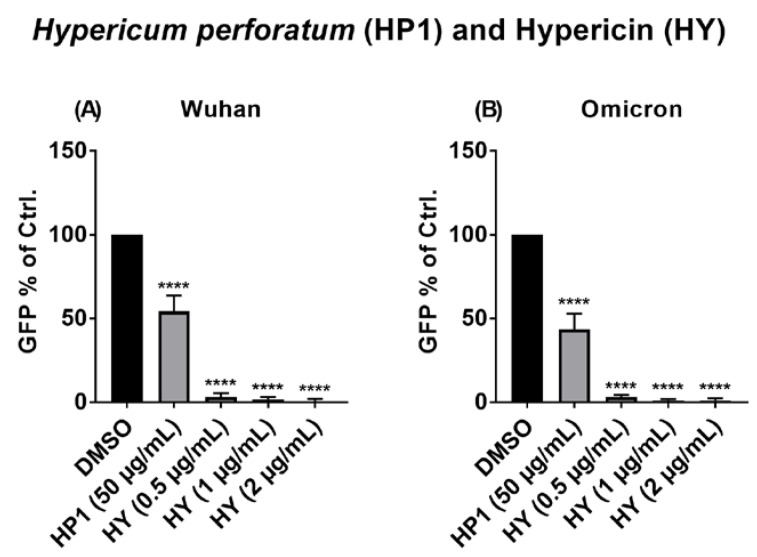
*Hypericum perforatum* (HP1) and its ingredient hypericin (HY) showed an antiviral capacity against the pseudotyped VSV virus carrying SARS-CoV-2 S protein of the Omicron variant. Vero cells were seeded overnight, and the day after, cells were treated for 1 h at 37 °C with fresh DMEM-10% FCS containing the solvent control (DMSO) or the indicated concentration of HP1 or hypericin. In parallel, the pseudo-typed virus carrying (**A**) the genuine SARS-CoV-2 S protein (Wuhan S protein sequence) or (**B**) the Omicron variant S protein (Omicron S protein sequence) was incubated with either solvent control or the indicated concentration of each substance at room temperature for 1 h. After the 1 h incubation, the virus solution (MOI = 0.01) was applied on cells for 1 h, at 37 °C for infection, followed by a DMEM-10% FCS wash step, and a final application of fresh DMEM-10 % FCS. On the next day, GFP-positive cells were counted by Celigo Image Cytometer (Nexcelom Bioscience, Lawrence, MA, USA). GFP-positive cells as % of control are shown (mean and s.d.), and one-way ANOVA with Dunnett’s multiple comparisons was done by comparing each value with the control. **** for *p* ≤ 0.0001.

As mentioned before, the ongoing SARS-CoV-2 pandemic has resulted in the emergence of different variants, some of which became dominant in their pattern of epidemiological circulation worldwide, fully displacing the previously prevalent strains. Consequently, testing *Hypericum perforatum* (HP1) and hypericin against the currently circulating SARS-CoV-2 variant (Omicron) was of major interest. Using a defined set of concentrations to be tested, the antiviral activity of HP1 and hypericin was analyzed against VSV pseudo-typed viruses carrying either the S protein of the genuine SARS-CoV-2 virus (Wuhan S protein sequence) or the Omicron variant (Omicron S protein sequence). As shown in Figure 7, the antiviral activity of both substances against the pseudo-typed VSV carrying the S protein of the Omicron variant could be confirmed and showed comparable efficiency as against the pseudo-typed virus carrying the S protein of the genuine Wuhan SARS-CoV-2 strain (Figure 7).

Taken together, our data clearly demonstrate a very strong antiviral activity of the *Hypericum perforatum* extract and its active ingredients against SARS-CoV-2, including multiple emerged variants. Mode of action analyses revealed a prominent interference in the very early phase of infection, potentially by a direct virus-infectivity-blocking activity of the tested compounds.

## 3. Discussion

Infections with SARS-CoV-2 can result in dramatically different outcomes ranging from asymptomatic to severe and even fatal. Vaccination against the virus is currently the most prevalent practice, which can prophylactically minimize disease severity but cannot completely stop virus transmission and incidences of disease [34]. Still, antiviral treatment interventions against the disease (COVID-19) are required for unvaccinated individuals or individuals suffering from vaccine-breakthrough infections. Many treatment options have been described to combat the disease, especially in hospitalized patients, where antivirals or remedy-assisting drugs are usually involved. Convalescent plasma [35] or monoclonal neutralizing antibody mixtures, such as Bamlanivimab, Etesevimab [36], and lately Xevudy (Sotrovimab) [37], remained as options of choice along with anti-inflammatory and/or immune-modulatory drugs, such as, e.g., the anti-JAK compound Baricitinib, the anti-IL6 compound Tocilizumab, and corticosteroids, as reported by Stebbing et al. [38], Jordan et al. [39], and Fernández-Cruz et al. [40], respectively, in addition to the anti-IL1 receptor antagonist Kineret (Anakinra) [41]. On the other hand, direct antivirals include inhibitors of essential processes in the virus life cycle, which are (i) spike maturation/fusion inhibitors (e.g., Camostat as anti-TMPRSS2 and Umifenovir), (ii) endosomal fusion inhibitors (e.g., Hydroxychloroquine and Azithromycin), (iii) protease inhibitors (e.g., Ritonavir, Lopinavir, and Darunavir), and (iv) polymerase inhibitors (e.g., Remdesivir and Favipiravir). Unfortunately, most of these drugs are non-SARS-CoV-2 specific, and many of them did not even show promising results in clinical trials or instead showed a high level of controversy among the different data sets. Suggested alternatives also included Fluoxetine, Plitidepsin, the MEK inhibitor ATR-002, Ivermectin, interferons, MAPK p38 inhibitors, and others [42,43]. Some of above-mentioned drugs successfully passed the designated steps of research and development. Nevertheless, others failed to pass, and many of them have an ill-defined mode of action. Thus, requirements for validation are still considered a must. By inducing SARS-CoV-2 mutagenesis and lowering the nasopharyngeal virus titers and the viral RNA, Molnupiravir (Merck) was able to pass the phase III clinical trial and gain authorization in the U.K. [44] but still does not have the marketing authorization granted by the European Medicines Agency (EMA). Trials on Paxlovid (Pfizer) as a specific SARS-CoV-2 3CL protease inhibitor allowed the drug to be authorized conditionally for commercial marketing [45]. Nevertheless, the need for SARS-CoV-2-directed drug therapies is still urgent due to their limited availability and/or accessibility.

In some countries, the usage of herbal medicine (medicinal plant extracts/ phytopharmaceuticals) is a common tradition in, e.g., veterinary interventions due to lack of financial resources and/or modern pharmaceuticals [46]. However, phytopharmaceuticals can gain governmental registration when the quality and clinical efficiency of the plant extract are demonstrated. In the early 2000s, some flavonoids from the seeds of *Aesculus chinensis* showed antiviral activity against respiratory syncytial virus (RSV), parainfluenza virus type 3 (PIV-3), and influenza virus type A (IAV), drawing more attention to their international recognition for use in humans [47]. Many natural compounds were proposed as antivirals against SARS-CoV-1 [48,49] and other viruses [50] and lately against SARS-CoV-2 (either as a direct treatment or as a recovery aid) [51,52]. However, such a practice has many arguments both for and against. Drawbacks include subjective efficacy, occasional acute poisoning or chronic toxicity, a slow mode of action, unknown source, unknown pharmacokinetic profile, unknown herbs–drug interactions, undefined active ingredients, poor regulatory measures, and frequent adulteration. Meanwhile, factors such as sustainability, accessibility, availability, affordability, multi-target effects, and easiness of uptake are considered points of advantage [53]. The analytical standardization and quantification of complex plant extracts can be easily and routinely performed by modern chromatographic methods.

In this study, initial trials revealed that the quantified *Hypericum perforatum* extract HP1, with its contents validated by chromatographic methods to confirm its naphtodianthrones, phloroglucinol, and flavonoid contents, inhibits infection by a pseudo-typed VSV virus that harbors the S protein of the SARS-CoV-2 virus (Figure S2), which was further confirmed by testing a range of *Hypericum perforatum* (HP1) concentrations (µg/mL) (Figure 1). As it is a simulation of SARS-CoV-2 S protein-mediated virus entry, the pseudo-typed virus system allowed us to identity *Hypericum perforatum* as an inhibitor that could abort the infection at its early stage, such as virus binding to cell receptors, fusion with host cells, uncoating inside virus-infected cells. Further assessments showed that the two naphtodianthrones (hypericin and pseudohypericin) are the most prominent active ingredients responsible for the antiviral activity against the pseudo-typed virus (Figure 2 and Figure 3), while other ingredients, such as the phloroglucinol derivative hyperforin, the condensed tannin procyanidin C1, and the flavonoid glycoside quercetin-3-*O*-glucuronid, were proven not to be active against SARS-CoV-2. (Figure 2). In line, it is well-established that hypericin is the major component of the *Hypericum perforatum* extract that generally inactivates a wide range of enveloped viruses [21]. Following the same direction, we could clarify that *Hypericum perforatum* and its ingredients hypericin and pseudohypericin were highly effective against the genuine infectious SARS-CoV-2 (Figure 4, Figures S4 and S5), where hypericin was the most active substance, as 25 ng/mL completely blocked virus replication. In line with the here-described antiviral properties of *Hypericum perforatum*, first indicative data about a potential antiviral activity of a commercial *Hypericum perforatum* extract, St. John’s wort, against SARS-CoV-2, are included in a not yet peer-reviewed manuscript by Bajrai et al. [54], although in this study, antiviral concentrations were close to toxic ranges of the extract.

Due to their high mutation rate, variants of SARS-CoV-2 have been and are still expected to emerge and may either spread all over the world, by displacing other variants, or disappear again. The frequent and ongoing emergence of SARS-CoV-2 variants dictates a critical overseen and evaluation of antiviral approaches against the emerging virus variants. Our investigations of antiviral activity of *Hypericum perforatum* and hypericin against the emerged B.1.1.7, B.1.351, and B.1.617.2 lineages revealed efficacy against the selected variants, which is relatively comparable to their effect against the ancestral SARS-CoV-2 (Figure 5). These data emphasize that the tested compounds seem to have a robust and broad antiviral activity, which is so far not affected by variant mutagenesis in the S-protein, data which we further confirmed by showing antiviral activity of HP1 and hypericin against the VSV pseudo-typed virus carrying the Omicron S protein (Figure 7).

To understand the antiviral mode of action of HP1 and its active ingredients, the pseudovirus system narrowed the options for tracing its possible mechanism of action, as it concerns mainly the early virus penetration events. In addition, our data using different treatment protocols are further indicative of a direct physical action of *Hypericum perforatum* and hypericin (as the most potent component) on virus particles due to their potent inhibitory effect on SARS-CoV-2, by only pre-treating the viruses with the substance prior to infection, seen in different experimental settings (Figure 6). Interestingly, the well-known S protein functions, such as ACE2 binding and cell fusion activity, are not affected by HP1 or hypericin (Figure S6).

Following the avenue of the mode of action investigations, our data therefore indicate that HP1 and hypericin directly affect SARS-CoV-2 virus particle infectivity, findings fitting to earlier reports of non-specific binding to viral membranes [22] or cross-linking between the substance and viral membrane proteins [55]. The description of membrane-binding capacities of these substances opens an intellectual door to think of blocking virus and host cell membrane fusion events as a mode of action of such compounds against viruses. Further supporting the idea of membranes being involved in the mechanism of virus blocking or even virucidal action, Tang et al. [56] suggested that the activity of hypericin is viral envelope-dependent, as hypericin was able to block propagation of multiple enveloped viruses but not of non-enveloped viruses (such as adenoviruses or polioviruses), when it was directly incubated with the virus. Of note, further cross-comparison investigations of membrane compositions of differently budding enveloped viruses, i.e., plasma membrane budding viruses such as influenza viruses [57] vs. intracellular compartment budding viruses, such as coronaviruses [58] or herpesviruses [59], and antiviral activities of, e.g., hypericin, against them could further clarify the detailed mechanism of action. Hypericin was also described as a protein–protein interaction inhibitor (PPI), as it can bind or change the localization of some cellular proteins/enzymes [60,61], similar to many PPIs such as erythrosine and others. This indicates that hypericin might have multimodal antiviral functions. However, upon taking the described information together, *Hypericum perforatum* and its active ingredients seem to have virus-binding and infectivity-blocking capacities. Furthermore, pre-treatments of cells do not block virus infectivity, arguing against a *Hypericum perforatum*-mediated blockade of the SARS-CoV-2 receptor ACE2. On the other hand, potential modes of antiviral actions of *Hypericum perforatum* against SARS-CoV-2 were introduced, since hypericin and pseudohypericin were anticipated, by molecular docking, to form a stable complex with the main (3CL) protease or the RNA polymerase of the SARS-CoV-2 [62,63] or had a high affinity toward SARS-CoV-2 basic proteins [64] but with only little clarification about biological evidence so far. However, it was found that some plant polyphenols could inhibit the 3CL protease using certain experimental settings [65]. Apart from the virological studies shown and discussed here, the well-described antidepressant effect of *Hypericum perforatum* was indirectly linked to its ability to inhibit synaptosomal reuptake of serotonin and other biogenic amines in vitro or to modulate serotonin receptors in vivo [66], suggesting that it may affect membranes (cellular or viral) in general. Depending on our previous assumptions of their mode of action and to complete the missing piece of the puzzle, further experiments are required to access the physicochemical interaction between *Hypericum perforatum* or its ingredients (mainly hypericin and pseudohypericin) and SARS-CoV-2 virus particles, with a special consideration to the viral membranes (i.e., envelope) [56]. In addition, the protein interactome of these substances inside cells and with SARS-CoV-2 proteins, in particular, should be further investigated. The multifactorial role of these substances is to be revealed by using targeted approaches in the future.

As regards the therapeutic usability of *Hypericum perforatum*, the extract is listed as a dietary supplement (with an attached mandatory disclaimer) in the USA but has not been approved by the FDA [67], and it is monographed by the European Medicine Agency EMA under the label of “well established use” for its anti-depressive activity. With being introduced thoroughly into several clinical trials [68,69], clinical development of a *Hypericum perforatum*-based treatment against SARS-CoV-2 would not start from scratch in respect to dose response, possible long-term supply, drug–drug interactions, and other factors. Interestingly, systemic bioavailability of hypericin from *Hypericum perforatum* extract after oral application has been well-documented within different clinical investigations [70,71]. From these data, the plasma levels of hypericin can be expected to be in the range of concentrations needed for inhibition of virus propagation. On the other side, the naphtodianthrones, as lipophilic compounds, are strongly albumin-bound in the plasma, which, however, is not expected to reduce its antiviral activity, as the protein-bound hypericin is in equilibrium with the free form [72,73]. To further emphasize its therapeutic potential in our in vitro approach, it was found that only post-treatment of cells with hypericin initiated after SARS-CoV-2 infection could clearly reduce the virus burden (Figure 6F), which may indicate a possible direct antiviral activity of hypericin against the newly produced and into the supernatant fluid secreted SARS-CoV-2 particles. Nevertheless, an intracellular mode of interference with virus propagation could potentially contribute to the observed antiviral activity.

Furthermore, concerning the putative usage of *Hypericum perforatum* as a treatment against SARS-CoV-2, hyperforin, another component of the *Hypericum perforatum* extract that did not act as an antiviral (Figure 2H), is assumed to control adverse inflammatory reactions and/or cytokine dysfunction in COVID-19 patients [74], making *Hypericum perforatum* to be potentially beneficial also in solving the immune deregulation-driven arm of the disease.

## 4. Materials and Methods

### 4.1. Cells

Baby hamster kidney (BHK-G43) cells were a kind gift from PD Dr. Gert Zimmer (Institute of Virology and Immunology, Mittelhäusern, Switzerland) and were kept in Glasgow’s Minimal Essential Medium (GMEM) supplemented with 5–10% fetal bovine serum (FBS). I1-Hybridoma cells (ATCC, CRL-2700) were kept in Dulbecco’s modified Eagle medium (DMEM-15% FBS) with 1% l-Glutamine, 100 IU/mL penicillin, and 0.1 mg/mL streptomycin. Human embryonic kidney (HEK293T) and African green monkey kidney (Vero E6) cells were maintained in DMEM-10 % FBS. All cells were incubated at 37 °C/5% CO_2_.

### 4.2. Compounds

*Hypericum perforatum* extract (HP1 dry extract batch 32700/M2) was kindly provided by Indena S.p.AS, Milan, Italy. As a dry extract, HP1 was obtained by a methanol-water extraction, which complies with the quality specifications of European Pharmacopoeia (Ed. 10) and contained 7.8% of flavonoids (HPLC), 4.4% of hyperforin (HPLCV), and 0.28% of total hypericins (HPLC) (INDENA, 2020; Certificate of Analysis-Personal Communication). Procyanidin-C1 and quercetin-3-*O*-glucuronid were isolated from *Hypericum perforatum* herbal material by the Institute of Pharmaceutical Biology and Phytochemistry at University of Muenster, Germany. Hypericin (#89226), pseudohypericin (#89261), and hyperforin (#89225) were purchased from Phytolab, Vestenbergsgreuth, Germany. All substances were dissolved in dimethyl sulfoxide (DMSO) to obtain stock concentrations of 10 mg/mL for HP1 and hypericin, 5 mM for procyanidin-C1 and quercetin-3-*O*-glucuronid, 5 mg/mL for hyperforin, and 1 mg/mL for pseudohypericin. After dissolving, substance preparations were aliquoted, stored at −20 °C, thawed immediately before each experiment under strongly reduced light exposure, and used as a single-use regiment.

For LC-DAD-ESI-qTOF-MS analysis of HP1, 10 mg of the dry extract were dissolved with 1000 µL of methanol and centrifugated. Then, 1 µL of this solution was injected into the LC-MS system. Chromatographic separations were performed on a Dionex Ultimate 3000 RS Liquid Chromatography System (Dionex, Rommerskirchen, Germany) on a Waters HSST3 column (2.1 × 100 mm, 1.7 µm) with a binary gradient (A: water with 0.1% formic acid; B: acetonitrile with 0.1% formic acid) at 0.4 mL/min: 0 to 0.4 min: linear from 5% B to 10% B, 0.4 to 6.1 min: linear from 10% B to 50% B, 6.1 to 8.1 min: linear from 50% B to 100% B, 8.1 to 15.0 min: isocratic at 100% B, 15.0 to 15.1 min: linear from 100% B to 5% B, 15.1 to 20 min: isocratic at 5% B. Eluted compounds were detected using a Dionex Ultimate DAD-3000 RS over a wavelength range of 200–400 nm, and a Bruker Daltonics micrOTOF-QII time-of-flight mass spectrometer equipped with an Apollo electrospray ionization source in positive mode at 3 Hz over a mass range of *m*/*z* 50–1500 using the following instrument settings: nebulizer gas nitrogen, 3 bar, dry gas nitrogen, 9 L/min, 200 °C, capillary voltage 4500 V, end plate offset −500 V, transfer time 70 µs, prepulse storage 5 µs, collision RF 100 Vpp, collision gas nitrogen. AutoMS² was set to a collision energy of 20 eV. Internal dataset calibration (HPC mode) was performed for each analysis using the mass spectrum of a 10 mM solution of sodium formiate in 50% isopropanol that was infused during LC re-equilibration using a divert valve equipped with a 20 µL sample loop. Data were analyzed using Bruker DataAnalysis 4.1 SP1.

### 4.3. Production of VSV-ΔG+G Virus

Selection of BHK-G43 was performed in GMEM-5% FBS containing 0.5 mg/mL Hygromycin B and 1 mg/mL Zeocin. Selected cells were stimulated to express the vesicular stomatitis virus (VSV)-G protein by addition of 1 nM Mifepristone into a fresh GMEM-5 % FBS (cells kept at 37 °C/5% CO_2_/6 h). Cells were then overnight infected with VSV-ΔG+G, which is a VSV virus that genetically lacks its G protein gene and contains a coding sequence for a green fluorescent protein (GFP) and luciferase (a kind gift from PD Dr. Gert Zimmer, Institute of Virology and Immunology, Mittelhäusern, Switzerland). Supernatants were collected, centrifuged at 200 g/5 min, aliquoted, and frozen at −80 °C. Later, the newly produced VSV-ΔG+G virus was titrated on Vero cells by serial dilution in DMEM-10% FBS and 1h infection at 37 °C in addition to 1× wash and 37 °C/5% CO_2_/16–18 h incubation in new DMEM-10% FBS media. GFP-positive cells were counted on the next day under the fluorescent microscope (Zeiss Axiovert 200M, Zeiss, Oberkochen, Germany) (titers expressed as fluorescent focus units per milliliter, FFU/mL).

### 4.4. C-Terminal Truncation of the Full-Length SARS-CoV-2 S Protein (d21)

The plasmid carrying the Wuhan SARS-CoV-2 full-length S protein sequence (YP_009724390.1, pCG1-SARS-2-S) was a kind gift of Prof. Dr. Stefan Pöhlmann (Infection Biology Unit, German Primate Center, Göttingen, Germany). The C-terminal truncation of the S protein of SARS-CoV-2 (i.e., deletion of the last 21 amino acids in the S protein) was executed to increase progeny virus titers, as this deletion results in (i) rapid trafficking of S protein to the cell surfaces and (ii) improved incorporation of the S protein into the VSV pseudovirus particles, which finally enhances virus yield titers [75]. The truncation was completed by Q5 site-directed mutagenesis kit (NEB, Ipswich. MA, USA, #E0552S) to introduce an additional stop codon that results in deletion of the last 21 amino acids of the S protein (tentatively named as d21). A pcDNA3.1(+) expression vector for expression of the SARS-CoV-2 Omicron variant S protein was designed (C-terminal truncated version lacking the last 21 amino acids “d21”) and subsequently synthesized by ThermoFisher, Waltham, MA, USA (GeneArt).

### 4.5. Production of the Pseudo-Typed VSV-ΔG SARS-CoV-2 S Protein (d21) Virus

We used a replication-defective/incompetent VSV virus, which carries the SARS-CoV-2 S protein on its surface as the sole glycoprotein used for the cell entry [76]. To produce this virus, on day 1, HEK-293T cells were seeded overnight in DMEM-10% FBS in poly-L-lysine-coated 10cm dishes. Cells were then supplied with fresh DMEM-10% FBS media. On day 2, pCG1-SARS-2-S d21 plasmid (Wuhan S protein sequence) or pcDNA3.1(+) SARS-CoV-2 Omicron variant S protein (Omicron S protein sequence) was transfected into these cells using an OptiMEM/TransIT-LT1 (Mirus, Madison, WI, USA, #MIR 2304,) transfection reagent mixture. On day 3, cells were infected with the VSV-ΔG+G virus in a DMEM-10% FBS infection mix for 1 h/37 °C. Afterwards, cells were 1× washed, incubated with anti-VSV G protein antibody (obtained from I1-Hybridoma cells) for 30 min at 37 °C (to neutralize potential still existing VSV-ΔG+G viruses), 2× washed, and incubated for 18–22 h at 37 °C (each step included fresh DMEM-10% FBS). On day 4, virus supernatants were collected, briefly centrifuged to remove cellular debris, introduced to a centrifugation-based concentration, using Amicon100 kD columns, (Merck, Darmstadt, Germany, #Z648043), and finally titrated on Vero cells to obtain FFU/mL titer. The obtained pseudo-typed virus (VSV-ΔG SARS-CoV-2 S protein d21) was used in infection models for the S protein-mediated entry of virus particles. If not otherwise stated in the figure legend, the Wuhan protein sequence for the S protein was used in experiments with the pseudo-typed particles.

### 4.6. Cell Cytotoxicity Assay (MTT Assay)

Vero cells were kept overnight in DMEM-10% FBS (96-well plate). On the next day, selected concentrations of each to-be-tested compound were dissolved in DMEM-10% FBS (the cell culture conditions of the pseudo-typed VSV-ΔG SARS-CoV-2 S protein d21 virus system) or infection-DMEM (DMEM supplemented with 1 mM sodium pyruvate, 1% non-essential amino acids “NEAA”, 10 mM HEPES, 2% FBS, 100 IU/mL penicillin, and 0.1 mg/mL streptomycin as the cell culture conditions of the SARS-CoV-2 infection system) to obtain the final indicated concentrations. Concurrently, the solvent control (DMSO) and the positive control (1 µM staurosporine, Sigma-Aldrich, Burlington, MA, United States, #S6942) were similarly prepared and applied on cells. Starting the incubation, old media were replaced by the fresh media containing DMSO or different concentrations of each compound or staurosporine, and cells were further incubated at 37 °C/5% CO_2_ for the designated time points. After incubation, 25 µL of fresh 5 mg/mL PBS-diluted MTT [3-(4,5-Dimethylthiazol-2yl)-2,5-diphenyl-tetrazolium-bromid] were applied directly to each well and incubated at 37 °C/ 5% CO_2_/1 h. Subsequently, MTT suspensions were removed, cells were lysed by adding 50 µL/well DMSO, and they were further incubated for 3–5 min at room temperature [77]. Finally, measurements were performed at 562 nm in a photometer, and the percentage (%) of cell survival was calculated in comparison to the solvent control DMSO.

### 4.7. SARS-CoV-2 Infection

The human SARS-CoV-2 viruses (i) hCoV-19/Germany/FI1103201/2020 isolate (EPI-ISL_463008) with the D614G mutation in its S protein, (ii) hCoV-19/Germany/NW-RKI-I-0026/2020 (B.1.1.7 “Alpha” variant), (iii) hCoV-19/Germany/NW-RKI-I-0029/2020 (B.1.351 “Beta” variant), and (iv) hCoV-19/Germany/326763/2021 (B1.617.2 “Delta” variant) were prepared at the Institute of Virology Muenster (IVM) by propagation on Vero cells (less than 5 passages).

The experimental protocol in this study involves the infection of Vero cells with corresponding SARS-CoV-2 at 37 °C/5% CO_2_/1 h in an infection phosphate-buffered saline (infection-PBS) mixture that includes PBS, 0.2% bovine serum albumin (BSA), 1 mM MgCl_2_, 0.9 mM CaCl_2_, 100 IU/mL penicillin, and 0.1 mg/mL streptomycin. Following infection, cells were washed with normal PBS and further incubated (up to 8 or 24 h) with infection-DMEM (with the same composition mentioned before).

### 4.8. Testing of Substances under Investigation against the Pseudo-Typed VSV-ΔG SARS-CoV-2 S Protein (d21) Virus or SARS-CoV-2 Virus

For the pseudo-typed SARS-CoV-2 S protein carrying VSV infection model, Vero cells were seeded overnight in DMEM-10 % FBS and kept at 37 °C/5% CO_2_ (96-well plate). Prior to infection, the protocol involved (a) pre-treatment of cells and (b) pre-treatment of the pseudo-typed virus, both with the substances under investigation. Therefore, cells were incubated at 37 °C/5% CO_2_/1 h in fresh DMEM-10% FBS media containing either solvent control (DMSO) or to-be-tested substances. Meanwhile, the pseudo-typed VSV-ΔG SARS-CoV-2 S protein-d21-carrying virus was incubated at room temperature/1 h in a DMEM-10% FBS infection mix containing the solvent control (DMSO) or to-be-tested substance. Following incubations, infection (MOI = 0.01) was executed at 37 °C/5% CO_2_/1 h. After infection, cells were washed and finally incubated with DMEM-10% FBS for 37 °C/5% CO_2_/18–22 h. After incubation, cells were visualized under the fluorescent microscope (unless otherwise stated in the figure legend) to count GFP-positive cells.

For the SARS-CoV-2 infection model, Vero cells were seeded in DMEM-10 % FBS and kept overnight at 37 °C/5% CO_2_ (12-well plate). The testing was performed, unless otherwise stated, by employing a treatment protocol, which includes (a) pre-treatment of cells, (b) pre-treatment of the virus, and (c) post-treatment of cells with the solvent control or to-be-tested substances. In brief, Vero cells were incubated at 37 °C/5% CO_2_/1 h with infection-DMEM (same composition as mentioned before) containing either solvent control or to-be-tested substance. Meanwhile, the SARS-CoV-2 virus was similarly incubated for 1h at room temperature (incubation was done in an infection-PBS mix “same composition as mentioned before”), that contains DMSO or the same concentration of the tested substance. Afterwards, the infection was performed at 37 °C/5% CO_2_/ 1 h with an MOI of 0.05 (unless otherwise stated), followed by a wash step with normal PBS. Finally, cells were further incubated for up to 24 h (unless otherwise stated) at 37 °C/5% CO_2_ with infection-DMEM (same composition as mentioned before) containing either solvent or to-be-tested substance.

### 4.9. Plaque Assay

For virus titration, supernatants were collected, frozen, and later used for standard plaque assay on Vero cells. Ten-fold serial dilutions of respective supernatants were prepared in infection-PBS (same composition as mentioned before) to infect cells (37 °C/5% CO_2_/1 h). After infection, inoculums were removed from cells and replaced with plaque MEM media containing 0.42% BSA, 1 mM l-glutamine, 20 mM HEPES, 0.24% NaHCO_3_, 200 IU/mL penicillin, 0.2 mg/mL streptomycin, 2% FBS, and 0.7% Oxoid agar and incubated at 37 °C/5% CO_2_/72–96 h. After removal of the agar, visible plaques were stained using Coomassie blue dye (Roth, Karlsruhe, Germany, Brilliant blue #R250), that dissolved in a methanol/acetic acid/distilled water mixture. Virus titers were calculated as plaque-forming units per milliliter (PFU/mL).

### 4.10. Indirect Immunofluorescence

Vero cells were seeded on glass coverslips (in 24-well plates). On the next day, SARS-CoV-2 virus was pre-treated with the solvent control (DMSO) or to-be-tested substances in an infection-PBS mixture (with the same composition mentioned before) for 1 h at room temperature. Afterwards, cells were virus-infected (MOI = 1) at 37 °C/1 h, washed with PBS, and further incubated in infection-DMEM (with the same composition mentioned before). Mock-infected cells served as control. At time points 2, 4, 6, and 8 h post infection, cells were fixed with −20 °C methanol at 4 °C/10 min and 1× washed with normal PBS. The staining procedures started with blocking with a 3% (*w*/*v*) bovine serum albumin (BSA) in PBS solution at room temperature/1 h. The SARS-CoV-2 Nucleocapsid Monoclonal Antibody (Invitrogen, Waltham, MA, USA, #MA5-29981) was diluted (1:1000) in 3% BSA-PBS and incubated with cells at room temperature for 1 h. Next, cells were 3× washed and then incubated with 3% BSA-PBS that contains 1:600 Alexa Fluor 488 secondary antibody (Invitrogen, Waltham, MA, USA, #A-10667) and 1:10,000 of 5 mg/mL 4’,6-Diamidino-2-Phenylindole, Dihydrochloride (DAPI, Invitrogen, Waltham, MA, USA, #D1306) at room temperature for 45 min in the dark. Finally, cells were washed 3× with PBS and 2× with ddH2O. The coverslips were mounted on glass slides by using fluorescent mounting medium (Agilent Dako, Santa Clara, CA, USA, #S3023). To capture and analyze pictures, the Axiovert 200 M microscope and AxioVision software V4.8.2.0 (Zeiss) were used.

### 4.11. hACE2-RBD Surrogate Virus-Neutralization Assay (sVNT)

For investigating the ability of the to-be-tested substances to inhibit the binding between human angiotensin converting enzyme 2 (hACE2) and the receptor binding domain (RBD) of the SARS-CoV-2 S protein, the sVNT cPass (Medac, Wedel, Germany) was used. The assay was performed according to the manufacturer’s manual. After test substances were solved in two-fold concentrations in sample dilution buffer, the same amount of RBD-HRP solution was added, and the samples were incubated for 30 min at 37 °C. From the mixture, 100 µL was added to a well of the ACE2 coated plate. The plate was sealed and incubated for 15 min at 37 °C. Solution was discarded from the wells, and every well was washed with 260 µL of wash solution four times. The substrate solution (100 µL) was added, and the plate was further incubated for 15 min in the dark at room temperature. The reaction was stopped by adding 50 µL of stop solution, and absorption was measured in a plate reader at 450 nm. Positive and negative control samples were supplied by the vendor.

### 4.12. Virus-Free Cell–Cell Fusion Assay

The SARS-CoV-2 S protein-mediated cell fusion activity was determined by using a Vero cell-based reporter enzyme assay containing the Tet-On 3G system. In brief, the stable cell lines Vero TRE3G-SEAP-EYFPNuc and Vero CMVTet3G were mixed in a ratio of 1:1, and 1.2 × 10^6^ cells were seeded per well (6-well culture plate). At 80–90% confluency, cells were transfected with 750 ng of pCG1-SARS-2-S plasmid expressing the SARS-CoV-2 S protein using Lipofectamine 2000 reagent (Invitrogen, Waltham, MA, USA). After 3.5 h, cells were washed with PBS once, detached with 0.3 mL trypsin/EDTA solution, and solved in a final volume of 1 mL of antibiotic-free MEM containing 10% FBS. Cells (7.5 × 10^4^) were seeded into each well of the 96-well plate, and test substances were added to reach concentrations as indicated to a final volume of 150 µL per well. Doxycycline hydrochloride solution (Merck, Darmstadt, Germany) was added to a final concentration of 10 µg/mL, and cells were incubated for 48 h at 37 °C/5% CO_2_. SEAP levels were determined with the Phospha-Light SEAP Reporter Gene System (Fisher Scientific, Schwerte, Germany) using 50 µL of supernatant and reagents. Measurements of luminescence (performed on white-bottom 96-well culture plates) were taken with an integration time of one second in a Glomax Explorer plate reader (Promega, Walldorf, Germany).

### 4.13. Statistical Analysis

All experiments were performed at least three independent times. Obtained data were analyzed by using GraphPad Prism (version 7) and finally represented as the mean and standard deviation (s.d.) of the independent experiments. Statistical significance (*p*-value) is labeled with stars (* for *p* ≤ 0.05, ** for *p* ≤ 0.01, *** for *p* ≤ 0.001, and **** for *p* ≤ 0.0001). Applied analyses (statistical tests) are indicated in each figure legend.

For the dose–response curves, obtained data were transformed to normalized values (%) of control and imported to the GraphPad Prism 7 software and the website: https://www.graphpad.com/quickcalcs/Ecanything1.cfm (accessed on 23 March 2022) to acquire the dose–response curves and the respective CC and IC values.

## 5. Conclusions

*Hypericum perforatum* and its ingredients, hypericin and pseudohypericin, act strongly antiviral against SARS-CoV-2 and several emerged variants. The blockade of virus propagation predominantly occurs at the very early stage of infection, presumably even at the level of interference with the virus particles, indicating a virucidal activity.

## Data Availability

Data is contained within the article and Supplementary Material.

## Supplementary Materials

The following supporting information can be downloaded at: https://www.mdpi.com/article/10.3390/ph15050530/s1, Table S1. Data of peaks identified by LC +ESI-qTOF-MS.; Table S2. Dose- response curve analysis of *Hypericum perforatum*, hypericin, and pseudohypericin against the pseudo-typed VSV virus carrying the SARS- CoV-2 S protein or the SARS-CoV-2 full virus; Figure S1: UV chromatograms of *Hypericum perforatum* (HP1) extract at λ = 360 nm (A) and 275 nm (B); Figure S2. *Hypericum perforatum* (HP1) acts antiviral against the pseudo-typed VSV virus; Figure S3: Structural features of natural products from *Hypericum perforatum* herbal material, included into functional assays; Figure S4. Hypericin showed a potent antiviral activity against SARS-CoV-2; Figure S5. Pseudohypericin possesses an antiviral activity against SARS-CoV-2; Figure S6. *Hypericum perforatum* (HP1) and its ingredient hypericin (HY) do not affect SARS-CoV-2 S protein-mediated ACE2 binding and fusion activity.
