# Supplementary material for: Hypericum perforatum and Its Ingredients Hypericin and Pseudohypericin Demonstrate an Antiviral Activity against SARS-CoV-2
Source: Pharmaceuticals (Basel). 2022 Apr 25;15(5):530. doi: 10.3390/ph15050530 (PMC9146521; doi:10.3390/ph15050530)
Supplement: Supplementary file 1 [file pharmaceuticals-15-00530-s001.zip › pharmaceuticals-1671550-supplementary.pdf]

pharmaceuticals

MDPI

Article

Hypericum perforatum and Its Ingredients Hypericin and Pseudohypericin Demonstrate an Antiviral Activity against SARS-CoV-2

Fakry F. Mohamed [1,6], Darisuren Anhlan [1], Michael Schöfbänker [1], André Schreiber [1], Nica Classen [2], Andreas Hensel [2], Georg Hempel [3], Wolfgang Scholz [4], Joachim Kühn [5], Eike R. Hrincius [1,†], and Stephan Ludwig [1,†,*]

1 Institute of Virology Muenster, Center for Molecular Biology of Inflammation (ZMBE), University Hospital Muenster, 48149 Muenster, Germany; framadan@uni-muenster.de (F.F.M.); anhlan@uni-muenster.de (D.A.); m_scho79@uni-muenster.de (M.S.); andre.schreiber@uni-muenster.de (A.S.); hrincius@uni-muenster.de (E.R.H.)
2 Institute of Pharmaceutical Biology and Phytochemistry, University of Muenster, 48149 Muenster, Germany; n_clas01@uni-muenster.de (N.C.); ahensel@uni-muenster.de (A.H.)
3 Division of Clinical Pharmacy, Institute of Pharmaceutical and Medical Chemistry, University of Muenster, 48149 Muenster, Germany; georg.hempel@uni-muenster.de
4 Hirsch Apotheke, 58507 Luedenscheid, Germany; wscholz@scholzdatabank.com
5 Division of Clinical Virology, Institute of Virology, University Hospital Muenster, 48151 Muenster, Germany; kuehnj@uni-muenster.de
6 Department of Virology, Faculty of Veterinary Medicine, Zagazig University, Zagazig 44511, Sharkia, Egypt
* Correspondence: ludwigs@uni-muenster.de
† These authors contributed equally to this work.

Supplementary materials:

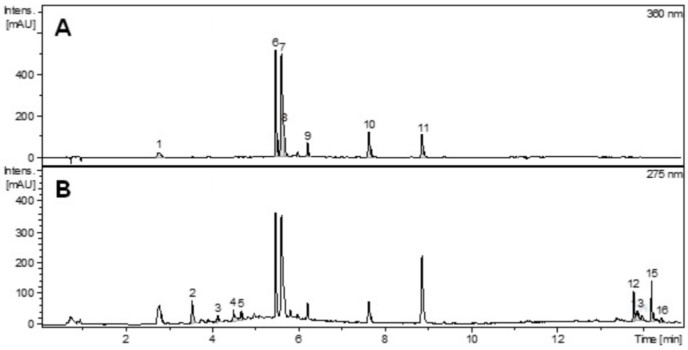

Figure S1: UV chromatograms of *Hypericum perforatum* (HP1) extract at λ = 360 nm (**A**) and 275 nm (**B**). Annotated peaks were identified by the accurate masses of the protonated molecules.

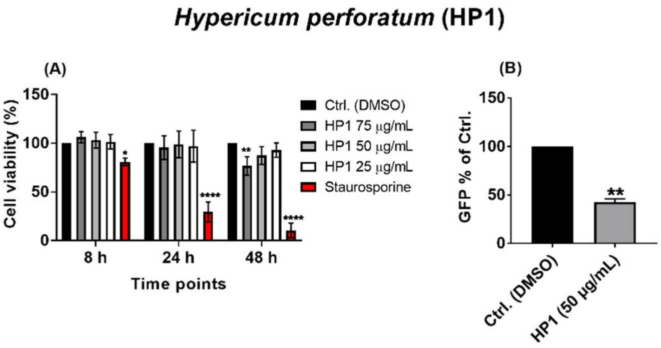

Figure S2. *Hypericum perforatum* (HP1) acts antiviral against the pseudo-typed VSV virus. **(A)** Vero cells were seeded overnight and at the next day incubation with *Hypericum perforatum* (HP1) or solvent control (DMSO) was initiated. Staurosporine treatment served as positive control. 8, 24 or 48 h after start of incubation, MTT assay-based cytotoxicity was measured, and cell viability as % of control is shown (mean and s.d.). Two-way ANOVA with Dunnett's Multiple comparisons was done by comparing each value with the control at each time point. **(B)** Vero cells were seeded overnight and on the next day, cells and the VSV-pseudo-typed virus were incubated with HP1 or solvent control (DMSO) for 1 h prior to infection, at 37 °C or room temperature, respectively. After pre-incubation, infection was performed with a MOI of 0.01 for 1 h and cells were finally washed and incubated without further treatments. After 16-18 h, GFP signal was visualized under fluorescent microscope. Data are shown as GFP positive cells as % of control (mean and s.d.) and students t-test with Welch's correction was applied. * for $p \leq 0.05$, ** for $p \leq 0.01$, and **** for $p \leq 0.0001$.

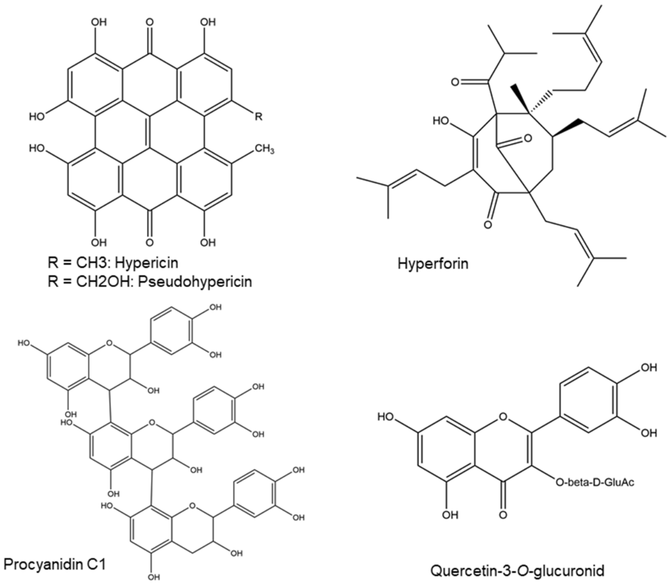

Figure S3: Structural features of natural products from *Hypericum perforatum* herbal material, included into functional assays.

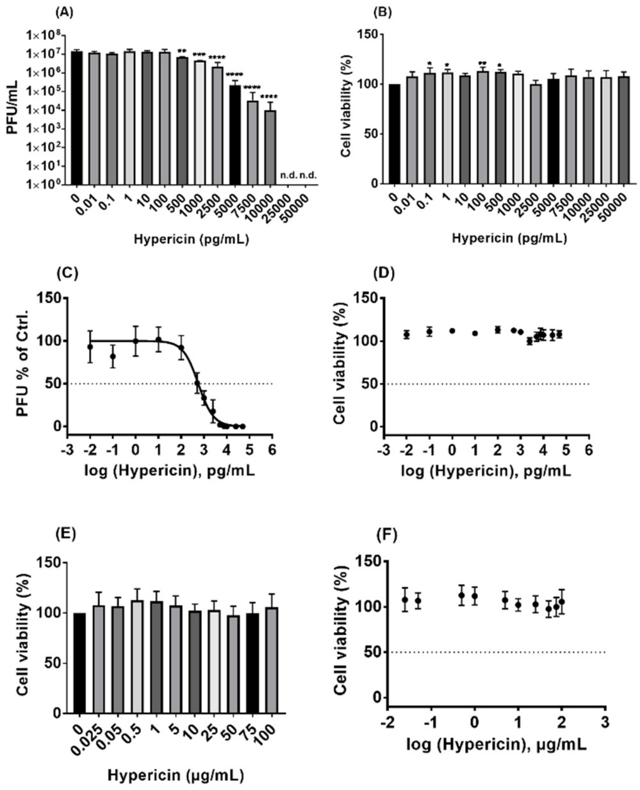

Figure S4. Hypericin showed a potent antiviral activity against SARS-CoV-2. **(A, C)** Vero cells were seeded overnight and on the next day, prior to infection (MOI =0.05), cells were incubated at 37 °C for 1 h with infection-DMEM containing either solvent control (DMSO) or hypericin. Concurrently, SARS-CoV-2 was incubated for 1 h at room temperature in infection-PBS that contains either DMSO or hypericin. After infection (37 °C/1 h), cells were further incubated in infection-DMEM including either DMSO or hypericin. After the 24 h infection, virus supernatants were collected and subjected to plaque assay. **(A)** Results are expressed as PFU/mL (mean and s.d.), and One-way ANOVA with Dunnett's multiple comparisons was done by comparing each value with the control. **(C)** Dose-response curve of the normalized virus titer values in % of control is depicted (mean and s.d.). **(B, D-F)** Vero cells were seeded overnight and on the next day, cells were incubated for 24 h with infection-DMEM that contains either solvent control (DMSO) or hypericin. After incubation, the MTT assay-based cytotoxicity was measured. **(B, E)** Cell viability as % of control is shown (mean and s.d.) and one-way ANOVA with Dunnett's multiple comparisons was done by comparing each value with the control. **(D, F)** Dose-response curve of the normalized cytotoxicity values in % of control is depicted (mean and s.d.). n.d. means non-detected. * for $p \leq 0.05$, ** for $p \leq 0.01$, *** for $p \leq 0.001$, and **** for $p \leq 0.0001$.

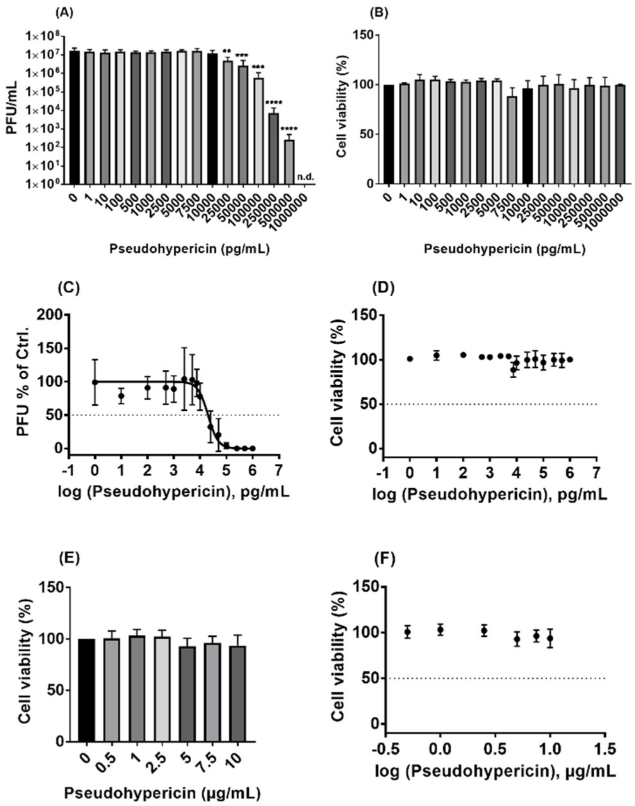

Figure S5. Pseudohypericin possesses an antiviral activity against SARS-CoV-2. **(A, C)** Vero cells were seeded overnight and on the next day, prior to infection (MOI = 0.05) cells were incubated at 37 °C for 1 h with infection-DMEM containing either solvent control (DMSO) or pseudohypericin. Concurrently, SARS-CoV-2 was incubated at room temperature for 1h in infection-PBS that contains either DMSO or pseudohypericin. After infection at 37 °C for 1 h, cells were further incubated in infection-DMEM including either solvent control or pseudohypericin. 24 h after infection, virus supernatants were collected and subjected to plaque assay. **(A)** The obtained results are expressed as PFU/mL (mean and s.d.), and One-way ANOVA with Dunnett's multiple comparisons was done by comparing each value with the control. **(C)** Dose-response curve of the normalized virus titer values as % of control is depicted (mean and s.d.). **(B, D-F)** Vero cells were seeded overnight and on the next day, cells were incubated for 24 h with infection-DMEM that contains either solvent control (DMSO) or pseudohypericin. After incubation, the MTT assay-based cytotoxicity was measured. **(B, E)** Cell viability as % of control is shown (mean and s.d.), and one-way ANOVA with Dunnett's multiple comparisons was done by comparing each value with the control. **(D, F)** Dose- response curve of the normalized cytotoxicity values as % of control is depicted (mean and s.d.). n.d means non-detected. ** for $p \leq 0.01$, *** for $p \leq 0.001$, and **** for $p \leq 0.0001$.

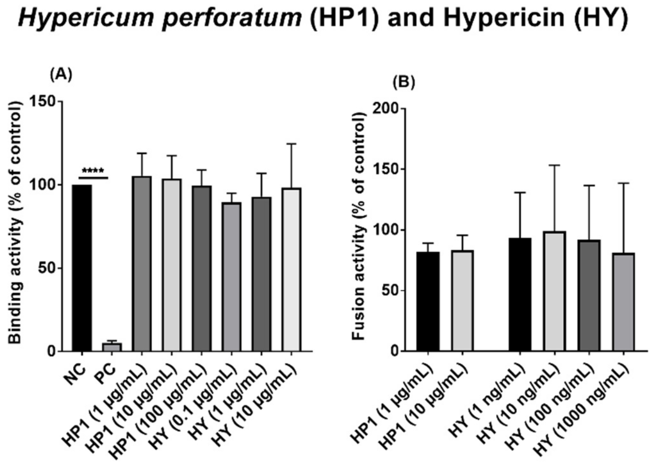

Figure S6. *Hypericum perforatum* (HP1) and its ingredient hypericin (HY) do not affect SARS-CoV-2 S protein-mediated ACE2 binding and fusion activity. (**A**) An hACE2 SARS-CoV-2 S protein RBD sVNT was conducted to test the ability of different concentrations of HP1 or hypericin to inhibit the specific binding of the SARS-CoV-2 S protein receptor binding domain (RBD) to hACE2. Data are represented as % of negative control (mean and s.d.). One-way ANOVA with Dunnett's multiple comparisons was done by comparing each value with the negative control. (**B**) A virus free cell-cell fusion assay was conducted to examine the fusion activity of the SARS-CoV-2 S protein upon treatment with different concentrations of HP1 or hypericin. In brief, to be fused, cells were transfected with SARS-CoV-2 expressing plasmid and treated with the indicated concentrations of HP1 and hypericin. Results are represented as SEAP reporter enzyme levels, which are correlated with the fusion activity (% of control) (mean and s.d.). **** for $p \leq 0.0001$.

Supplementary Table S1. Data of peaks identified by LC +ESI-qTOF-MS.

Peak No.	t_R/min	Identity	*m*/*z* [M+H]$^+$	Ion formula	err/mDa	mSigma
1	2.8	Monocaffeoyl quinic acid	355.1029	$[C_{16}H_{19}O_9]^+$	0.5	25.1
2	3.5	Monocumaroyl quinic acid	339.1097	$[C_{16}H_{19}O_8]^+$	2.3	7.8
3	4.1	Dimeric B-type procyanidin	579.1551	$[C_{30}H_{27}O_{12}]^+$	-5.4	11.7
4	4.5	(Epi)catechin	291.0899	$[C_{15}H_{15}O_6]^+$	3.6	7.4
5	4.7	Procyanidin C1	867.2232	$[C_{45}H_{39}O_{18}]^+$	–10.1	48.7
6	5.5	Rutin	611.1651	$[C_{27}H_{31}O_{16}]^+$	4.5	1.5
7	5.6	Hyperoside	465.1061	$[C_{21}H_{21}O_{12}]^+$	3.3	3.8
8	5.6	Isoquercitroside	465.1052	$[C_{21}H_{21}O_{12}]^+$	2.4	16.9
		Quercetin-3-*O*-glucuronide	479.0864	$[C_{21}H_{19}O_{13}]^+$	4.4	11.9
9	6.2	Quercitrin	449.1099	$[C_{21}H_{21}O_{11}]^+$	2.1	29.4
10	7.6	Quercetin	303.0514	$[C_{15}H_{11}O_7]^+$	1.5	13.5
11	8.9	Biapigenin	539.1032	$[C_{30}H_{19}O_{10}]^+$	5.9	8.3
12	13.8	Unidentified Phloroglucinol	553.3938	$[C_{35}H_{53}O_5]^+$	–5.1	38.3
13	13.9	Unidentified Phloroglucinol	553.3936	$[C_{35}H_{53}O_5]^+$	4.8	20.2
14	14.0	Unidentified Phloroglucinol	567.4068	$[C_{36}H_{55}O_5]^+$	–2.4	32.3
15	14.2	Hyperforin	537.3979	$[C_{35}H_{53}O_4]^+$	4.0	14.0
16	14.4	Adhyperforin	551.4128	$[C_{36}H_{55}O_4]^+$	3.3	9.9

Supplementary Table S2. Dose-response curve analysis of *Hypericum perforatum*, hypericin, and pseudohypericin against the pseudo-typed VSV virus carrying the SARS- CoV-2 S protein or the SARS-CoV-2 full virus

Substance	IC50 *	CC50 *	Selectivity index ($SI=CC_{50}/IC_{50}$)
**Pseudo-typed virus system:**			
Hypericum perforatum	36.88 μg/mL	>100 μg/mL	>2.71
Hypericin	48.57 ng/mL	>1000 ng/mL	>20.59
Pseudohypericin	298.4 ng/mL	>2000 ng/mL	>6.7
**SARS-CoV-2 virus system:**			
Hypericum perforatum	1.353 μg/mL	>100 μg/mL **	>73.91
Hypericin	559.1 pg/mL	>100 μg/mL **	>178858.88
Pseudohypericin	20036 pg/mL	>10 μg/mL **	>499.1

* Normalized values have been used for calculations

** Escalated CC50 (highest possible concentration tested for toxicity)
